# Losing without Fighting - Simple Aversive Stimulation Induces Submissiveness Typical for Social Defeat via the Action of Nitric Oxide, but Only When Preceded by an Aggression Priming Stimulus

**DOI:** 10.3389/fnbeh.2017.00050

**Published:** 2017-03-22

**Authors:** Jan Rillich, Paul A. Stevenson

**Affiliations:** Institute for Biology, Leipzig UniversityLeipzig, Germany

**Keywords:** association, biogenic amines, decision-making, experience-dependent-plasticity, insects, neuromodulation, priming, submissive behavior

## Abstract

Losing a fight (social defeat) induces submissiveness and behavioral depression in many animals, but the mechanisms are unclear. Here we investigate how the social defeat syndrome can be established as a result of experiencing aversive stimuli and the roles of neuromodulators in the process. While biogenic amines and nitric oxide (NO) are associated with reduced aggression in mammals and insects, their specific actions during conflict are unknown. Although the social defeat syndrome normally results from complex interactions, we could induce it in male crickets simply by applying aversive stimuli (AS) in an aggressive context. Aggressive crickets became immediately submissive and behaved like losers after experiencing two brief AS (light wind puffs to the cerci), but only when preceded by a priming stimulus (PS, stroking the antenna with another male antenna). Notably, submissiveness was not induced when the PS preceded the AS by more than 1 min, or when the PS followed the AS, or using a female antenna as the preceding stimulus. These findings suggest that any potentially detrimental stimulus can acquire the attribute of an aversive agonistic signal when experienced in an aggressive context. Crickets, it seems, need only to evaluate their net sensory impact rather than the qualities of a variety of complex agonistic signals. Selective drug treatments revealed that NO, but not serotonin, dopamine or octopamine, is necessary to establish the submissive status following pairing of the priming and aversive stimuli. Moreover, treatment with an NO donor also induced the social defeat syndrome, but only when combined with the PS. This confirms our hypothesis that aversive agonistic experiences accumulated by crickets during fighting invoke social defeat *via* the action of NO and illustrates that a relatively simple mechanism underlies the seemingly complex social decision to flee. The simple stimulus regime described here for inducing social defeat opens new avenues for investigating the cellular control of subordinate behavior and post-conflict depression.

## Introduction

Aggressive competition between animals of the same species is a widespread behavioral strategy for securing limited resources (Nelson, 2006). However, in the face of the inherent risks, animals must in some way know when it would be more opportune to flee rather than persist in fighting (Hardy and Briffa, 2013). At present, knowledge of the effects of diverse neurotransmitters, modulators and hormones on aggression in classical “model” species is rapidly growing (rodents: Nelson and Trainor, 2007; cichlid fish: Oliveira et al., 2009; *Drosophila*: Kravitz and Fernandez, 2015; Hoopfer, 2016). Despite this, however, the proximate mechanisms that enable animals to actually weigh up the odds for the decision to fight or flee are largely unknown. The decision to flee is generally thought to be based on the assessment of agonistic signals exchanged during fighting (Hurd, 2006). Several theoretical models for this have been proposed, which differ largely with respect to whether the contesting individuals are considered to assess only their own, their opponent's, or compare each other's agonistic signals (Payne, 1998; Hurd, 2006). Currently, debate continues on whether animals, particularly invertebrates, possess the level of cognitive capacity required by such models for assessing agonistic signals (Elwood and Arnott, 2012, 2013; Fawcett and Mowles, 2013).

At present, this topic is probably better understood in crickets, which exhibit spectacular fighting behavior (reviews: Stevenson and Rillich, 2016, 2017). In these insects, the decision to fight is promoted by octopamine (the invertebrate analog of noradrenalin). This biogenic amine has been shown to mediate the aggression enhancing effects of a wide variety of experiences including physical exertion (e.g., flying: Stevenson et al., 2000, 2005), the possession of resources (e.g., a burrow: Rillich et al., 2011) and winning a fight (Rillich and Stevenson, 2011). Octopamine is thus considered to represent the motivational component of aggression (Stevenson and Rillich, 2016, 2017). The basic strategy used by crickets for timing the decision to flee, on the other hand, was revealed by manipulating agonistic signals exchanged during fighting (Rillich et al., 2007). Crickets conform to the Cumulative Assessment Model proposed by Payne (1998), in that they assess only their opponent's agonistic actions and flee the moment the sum accrued during a contest exceeds some critical amount (Rillich et al., 2007). More recently it was revealed that the influence of an opponent's agonistic signaling efforts is mediated by the nitric oxide/cyclic guanosine 3′, 5′-monophosphate (NO/cGMP) pathway, which in effect acts to promote the decision to flee (Stevenson and Rillich, 2015). In mammals, disruption of genes encoding nitric oxide synthase (NOS) is also associated with increased aggression (Nelson and Trainor, 2007), but the specific behavioral role of NO in aggression is not known. In crickets, the data suggest that the actions of an opponent experienced during fighting leads to activation of NOS. NO production then acts to induce a state of behavioral submissiveness (Stevenson and Rillich, 2015), which is typical for losers and generally lasts at least 3 h (Stevenson and Rillich, 2013). The experience of losing, is associated with prolonged submissiveness in many animals (Hsu et al., 2006). This so called social defeat syndrome is also regarded as a major stressor which plays a role in psychiatric disorders such as depression and post-traumatic stress disorder (Huhman, 2006; Hollis and Kabbaj, 2014). Accordingly, there is much interest in discovering its proximate cause, which is currently unknown in mammals.

Here we test the hypothesis that the behavioral syndrome of social defeat can be induced, without actually losing a fight, simply by applying aversive stimulation (AS) to activate NOS. We have previously shown that brief wind stimulation of the abdominal cerci induces submissiveness in aggressive crickets that had just won a previous fight (Stevenson and Rillich, 2015). Importantly, this was achieved at a stimulus intensity far below that required to induce escape behavior (cf. Gras and Hörner, 1992; Stevenson et al., 2005; Oe and Ogawa, 2013; Fukutomi et al., 2015). Furthermore, the AS was only effective when applied immediately after winning, but not when delivered 3 min later. Hence, we investigate whether or not socially naïve crickets respond to AS only when experienced in an aggressive context, i.e., after interacting with another male. We attempt this by evaluating the efficacy of the AS in combination with stimulation using another male's antenna, to mimic antennal fencing between crickets that occurs at the start of a fight. Lashing a male cricket's antenna with an antenna from another conspecific male evokes the mandible threat response, which ceases when stimulation stops, and is otherwise seen only during escalating fighting (e.g., Alexander, 1961; Adamo and Hoy, 1995; Hofmann and Schildberger, 2001). It is thus considered to be the natural releasing stimulus for aggression. Stimulation with a male antenna also has a longer lasting effect in that it increases aggression of stimulated crickets at contests staged 10 min later (Rillich and Stevenson, 2015). Notably, this enhancing effect is not clear in fight-inexperienced crickets, which are normally highly aggressive anyway, but dramatic in losers which are normally submissive. Stimulation with a male antenna thus fulfills the definition of a priming stimulus (PS, cf. Schacter et al., 2004) for cricket aggression. Finally, using selective drugs we tested whether the effects of AS and PS depends on NO or the amines octopamine, dopamine and serotonin, which also influence insect aggression (crickets: Stevenson and Rillich, 2016; *Drosophila* Hoopfer, 2016) and aversive associative learning (crickets: Unoki et al., 2005; Matsumoto et al., 2013; *Drosophila*: Schwaerzel et al., 2003).

## Materials and methods

### Experimental animals

Mature, 2–3 week old, adult male Mediterranean field crickets, *Gryllus bimaculatus* (de Geer) were taken from a breeding stock maintained under constant standard conditions at Leipzig University (22–24°C, relative humidity 40–60%, 12 h: 12 h light: dark regime daily feeding on bran and fresh vegetables). All experiments were performed during daylight hours, avoiding times when aggression tends to be depressed (just after midday and on generally dreary days; cf. Stevenson et al., 2000). All animal treatments complied with the Principles of Laboratory Animal Care and the German Law on the Protection of Animals (Deutsches Tierschutzgesetz).

### Evaluation of aggression

Unless stated otherwise, all tested crickets were mature adult males that had no social contact to conspecifics for at least 24 h (“naive”), after which all known effects of previous social interactions on aggressive behavior have abated (Stevenson and Rillich, 2013). The aggressive behavior of test crickets that received various pre-treatments (see below) was evaluated by matching them in dyadic contests against equally sized males (<5% weight difference) that were induced to be hyper-aggressive by flying them in a wind stream for 3 min shortly before the match (cf. Hofmann and Stevenson, 2000). Since in these experiments the hyper-aggressive crickets always won the contest, they served as a standard, against which the aggressiveness of test crickets could be directly compared in dyadic contests (see also Stevenson and Rillich, 2013, 2015).

For each test, a pair of crickets were placed at opposite ends of a clear Perspex-glass rectangular fighting arena (l. w. h.: 16 × 9 × 7 cm) with a sand-covered floor divided halfway along its length by an opaque sliding door. On removing the door, the animals' interactions follow a stereotyped sequence typical for fights in the field (Alexander, 1961) which we score on a scale of 0-6 to denote aggressive escalation (Hofmann and Stevenson, 2000; Stevenson et al., 2000): Level 0: mutual avoidance without aggression. Level 1: one cricket attacks, the other retreats. Level 2: antennal fencing. Level 3: mandible spreading by one cricket. Level 4: mandible spreading by both crickets. Level 5: mandible engagement. Level 6: grappling, an all-out fight. Contests can finish at any level with the retreat of one opponent, and fight duration was measured to the nearest second with a stopwatch, deducting pauses that occasionally occurred when the animals lost contact. Since the hyper-aggressive individual always won, the level of aggression gives the level to which it had to escalate in order to induce the test cricket to retreat.

### Treatments of test crickets before fighting

#### Priming stimulus (PS)

As in our earlier study (Rillich and Stevenson, 2015) freshly excised antennae from a mature, adult male donor cricket was used to stroke the test cricket's antennae continually for 30 s. The effect of this on aggression was tested 10 min later. In control experiments, we tested the effects of using female antenna and male antennae that were washed twice for 10 min with n-hexane to remove cuticular pheromones (Iwasaki and Katagiri, 2008).

#### Aversive stimulus (AS)

As in our previous study (Stevenson and Rillich, 2015), we used a remotely controlled two-way valve (Lee, Conn., USA) connected to a compressed air supply to deliver 1–4 brief wind puffs (200 ms, 1 s intervals) to a cricket's abdominal cercal appendages from a hand-held glass tube (5 mm diameter, 7–14 cm distant). Wind velocity was set such that 4 stimuli just evoked a noticeable motor response involving 1–2 steps at most. This was achieved with a mean velocity measured with an anemometer at the tube opening of 3.8 m/s (standard error 0.37, *n* = 15). This results in a mean stimulus velocity measured at the cercus of 0.36 m/s (standard error 0.06, *n* = 15). With this regime we never observed walking sequences, escape runs or jumps, which often occur at higher stimulus intensities (Gras and Hörner, 1992; Stevenson et al., 2005; Oe and Ogawa, 2013; Fukutomi et al., 2015).

#### Pharmacological treatments

All drugs were obtained from Sigma Aldrich (Deisenhofen, Germany). Depending on solubility, drugs were dissolved either in insect saline (contents in mM: NaCl 140, KCl 10, CaCl_2_ 7, NaHCO_3_ 8, MgCl_2_ 1, N-trismethyl-2-aminoethanesulfonic acid 5, d-trehalose dihydrate, pH 7.4) or first in dimethylsulfoxide (DMSO) and subsequently diluted in insect saline to give the required drug concentration in 1% DMSO as vehicle. Using a micro-syringe (Hamiliton®, Bonaduz, Switzerland), drug solutions were injected into haemocoel *via* the pronotal shield, in preference to the head capsule, since in our hands we then occasionally observed detrimental effects on behavior with vehicle. The selectivity of used drugs and their most effective dosages that influenced cricket aggressive behavior, without any obvious detrimental effect on general motility, has been determined in prior investigations (Stevenson et al., 2005; Rillich and Stevenson, 2011, 2014, 2015; Rillich et al., 2011; Stevenson and Rillich, 2015, see also Figures S1, S2). On the basis of these earlier findings, we evaluated the aggressive behavior of test crickets 30-60 min after a single 20 μl injection of the following: The selective octopamine-receptor (OAR) blocker epinastine hydrochloride (10 mM in 1% DMSO; see also Roeder et al., 1998), the insect dopamine-receptor (DAR) blocker fluphenazine dihydrochloride (10 mM in 1% DMSO; see also Degen et al., 2000), a cocktail of the serotonin receptor (5HTR) blockers methiothepin mesylate salt and ketanserin (+)-tartrate salt (10 mM of each in 1% DMSO), which together should block all known insect 5HT receptors (cf. Tierney, 2001; Anstey et al., 2009; Wright et al., 2010; Thamm et al., 2013), the competitive nitric oxide (NO) synthase inhibitor N_ω_-Nitro-L-arginine methyl ester hydrochloride (LNAME) or its non-effective enantiomer DNAME as control (10 mM, each in insect saline), the nitric oxide donor S-Nitroso-N-acetyl-DL-penicillamine (SNAP, 1 mM in insect saline). Drug dosages in μg and μg/g body weight are given in Table S1. The effect of each drug was compared to that of the corresponding vehicle, in separate groups of control animals for each drug, that were injected and tested at the same time as test animals.

### Data analysis

All statistical tests were performed using Prism 6 (GraphPad Software Inc., La Jolla, CA, USA) running on a Macintosh computer (Apple Computers, Cupertino, CA, USA). The median and the interquartile range (IQR) were calculated for non-parametric data sets. Non-parametric tests were also performed on duration since the data sets failed D'Agostino and Pearson omnibus normality tests, even after log transformations. The Mann-Whitney U-test was used to test for significant differences in the distributions between unpaired data sets. In experiments in which multiple groups were compared we used the Kruskal-Wallis test with Dunn's multiple comparisons test. The Chi-square test was used to compare the relative frequencies of level 1 fights (immediate retreats). Each test cricket was used for only one experiment, and the numbers used for each experimental test group are indicated in the figures and tables.

## Results

### Aversive stimulation (AS) and aggression in socially naive crickets

**Figure 1:**
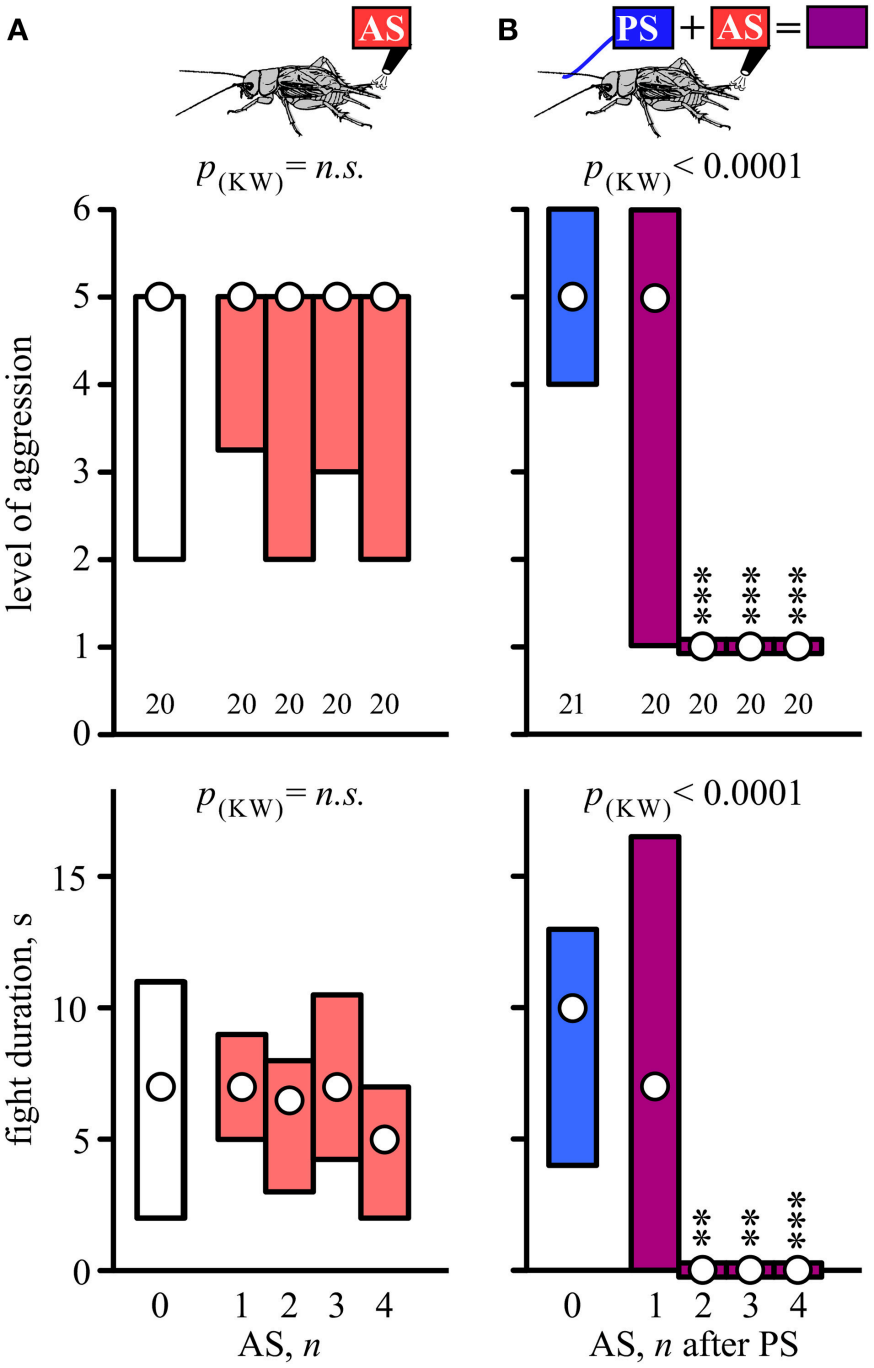
**Setting social status by prior stimulation. (A)** Aggressiveness of crickets matched against a standard hyper-aggressive opponent 10 min after different stimuli (top, stimulus regime; middle, level of aggression; bottom, fight duration). Circles: median, boxes: interquartile range, *n* is given below the bars. The animals received either no stimulation (0, white bars) or aversive stimulation (AS, red bars: 1–4 wind puffs at 1s intervals). **(B)** As in A for crickets that received either the priming stimulus alone (PS, blue bars) or the PS followed by 1–4 AS at 1 s intervals (purple bars). Significant differences are indicated above the bars by *p*-value from Kruskal-Wallis tests (p_(*kw*)_) and asterisks for Dunn's multiple comparisons test compared to PS only in **(B)** (^**^p < 0.01, ^***^p < 0.001).

Socially naive crickets that received no stimulation usually escalated against the flown “standard hyper-aggressive” opponents to the physical level of mandible engagement (median level 5, IQR 2-5, *n* = 20) in fights that lasted 2–11 s (IQR, median 7 s; Figure 1A, white bars). Compared to this, aggressive behavior was not affected by prior aversive stimulation (AS) applied on its own 10 min beforehand (Figure 1A, red bars). Altogether we tested the effects of 1, 2, 3, and 4 AS, delivered at 1 s intervals on groups of 20 naive crickets and found no statistically significant influence of prior AS on aggression (Kruskal-Wallis test: level, *p* = 0.7119; duration, *p* = 0.4471; Figure 1A, Table 1).

**Table 1:** **Table giving fight level and duration of aggression for fights of test crickets against standard hyper-aggressive opponents: median (50%), IQR (25, 75%), minimum (min.), maximum (max.), sample size (*n*) and statistics values from Kruskal-Wallis tests (*H, p*)**.

	**Group**	**Fight level**						**Fight duration, s**					**Statistics**	
		**n**	**min**	**25%**	**50%**	**75%**	**max**	**min**	**25%**	**50%**	**75%**	**max**	***H***	***p***
A	0AS	20	1	2	5	5	6	0	2	7	11	18	Level:	
	1AS	20	1	3.25	5	5	6	0	5	7	9	52	2.13	0.712
	2AS	20	1	2	5	5	6	0	3	6.5	8	17	Duration:	
	3AS	20	1	3	5	5	6	0	4.25	7	10.5	36	3.71	0.447
	4AS	20	1	2	5	5	5	0	2	5	7	12		
B	PS	21	1	4	5	6	6	0	4	10	13	52	Level:	
	PS+1AS	20	1	1.5	4	5	6	0	0.5	7	16.5	32	54.12	0.0001
	PS+2AS	20	1	1	1	1	5	0	0	0	0	6	Duration:	
	PS+3AS	20	1	1	1	1	5	0	0	0	0	7	53.69	0.0001
	PS+4AS	20	1	1	1	1	4	0	0	0	0	5		

***(A)** Data for test crickets that received 0–4 aversive stimuli (AS) prior to fighting. **(B)** Data for test crickets that received priming stimulus alone (PS) or PS paired with 1–4 AS. The data are depicted in Figure 1*.

### Aversive stimulation (AS) paired with the aggression-priming stimulus (PS)

Contrasting the above, AS applied 1 s after a delivery of an aggression-priming stimulus (PS; stroking the antenna with another male antenna), significantly suppressed the expression of aggression in fights staged 10 min subsequently (Figure 1B). As shown previously for fight-inexperienced crickets (Rillich and Stevenson, 2015), the PS on its own did not significantly change the expression of aggression exhibited 10 min later in fights against the standard hyper-aggressive opponents (median level 5, IQR 4-6, *n* = 21, median duration 10 s, IQR 4–13; Figure 1B, blue bar). However, paired stimulation, in which the PS directly preceded 1–4 AS significantly suppressed aggressiveness (Kruskal-Wallis-test: level, *p* < 0.0001; duration, *p* < 0.0001; Figure 1B, purple bars, Table 1). This effect depended on the number of applied AS (Figure 1B). Whereas the crickets still escalated against standard hyper-aggressive opponents after receiving the PS and only one AS (median level 5, IQR 1–6, *n* = 20), nearly all behaved submissive after receiving 2 AS (median level 1, IQR 1–1, *n* = 20; 85% level 1) or more after the PS. Importantly, this aggression suppressing effect of paired stimulation did not depend on whether the animals previously responded to the PS with or without the threat display prior to testing their aggressiveness. Taking all data for PS paired with 2, 3, and 4 AS into account (*n* = 60, Figure 1B), 47% exhibited the threat display on receiving the PS, of which 82% exhibited immediate retreat (level 1) on confronting the hyper-aggressive opponent 10 min later, compared to 88% immediate retreats for individuals that did not previously respond to the PS with threat display (not significantly different: Chi-square test, Chi-value = 0.426, *p* = 0.514). In both cases the frequency of immediate retreats is significantly greater than for untreated crickets (15%, *n* = 20, Chi-square test compared to with and without threat display: Chi-value = 21.1, *p* < 0.001 respectively Chi-value = 27.3, *p* < 0.001).

**Figure 2:**
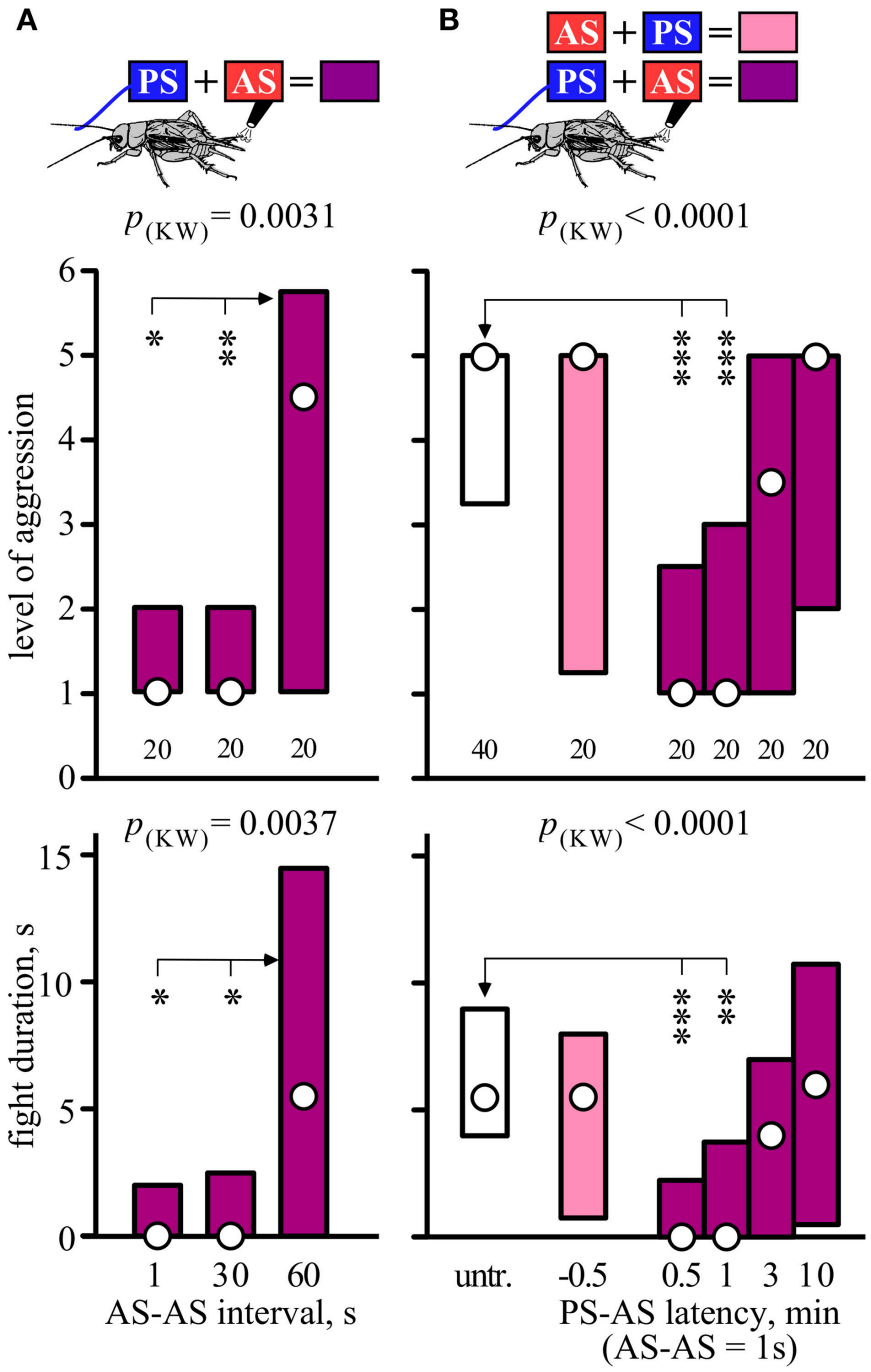
**Significance of AS-AS interval and PS-AS latency. (A)** Effect of increasing the interval between two consecutive AS (1, 30, and 60 s), preceded by the PS, on the aggressiveness of crickets matched against a standard hyper-aggressive opponent 10 min after stimulation (top, stimulus regime; middle, level of aggression; bottom, fight duration). Circles: median, boxes: interquartile range, *n* is given below the bars. **(B)** Effect of PS-AS latency. The animals were either untreated (white bars) or received the PS and 2AS, whereby the AS were delivered either before the PS (−0.5 min, pink bars) or after the PS (0.5, 1, 3, or 10 min, purple bars). Significant differences are indicated by *p*-values from Kruskal-Wallis tests (p_(kw)_ in **A,B**) and asterisks for Dunn's multiple comparisons test compared to AS-AS interval 60 s in **(A)** and untreated in **(B)** (^*^p < 0.05, ^**^p < 0.01, ^***^p < 0.001).

The effectiveness of paired stimulation also depended on the interval between the two stimuli (Figure 2A). While the suppressing effect of 2 AS spaced 1 or 30 s apart were equally effective, an interval of 60 s between them no longer suppressed aggressiveness (Kruskal-Wallis-test: level, *p* = 0.0031, duration, *p* = 0.0037; Figure 2A, Table 2).

**Table 2:** **Table giving fight level and duration of aggression of aggression for fights of test crickets against standard hyper-aggressive opponents: median (50%), IQR (25, 75%), minimum (min.), maximum (max.), sample size (*n*) and statistics values from Kruskal-Wallis tests (*H, p*)**.

	**Group**	**Fight level**						**Fight duration, s**					**Statistics**	
		**n**	**min**	**25%**	**50%**	**75%**	**max**	**min**	**25%**	**50%**	**75%**	**max**	***H***	***P***
A	PS+2AS/1s	20	1	1	1	2	5	0	0	0	2	7	Level:	
	PS+2AS/30s	20	1	1	1	2	5	0	0	0	2.5	12	11.53	0.0031
	PS+2AS/60s	20	1	1	4.5	5.75	6	0	0	6	14.5	42	Duration:	
													11.23	0.0037
B	untreated	40	1	3.25	5	5	6	0	4	5.5	9	45		
	2AS+PS/0.5min	20	1	1.25	5	5	6	0	0.75	5.5	8	18	Level:	
	PS+2AS/0.5min	20	1	1	1	2.5	6	0	0	0	2.25	6	31.4	0.0001
	PS+2AS/1min	20	1	1	1	2.75	6	0	0	0	2.75	10	Duration:	
	PS+2AS/3min	20	1	1	3.5	5	6	0	0	4	7	9	35.1	0.0001
	PS+2AS/10min	20	1	2	5	5	6	0	0.5	6	10.75	21		

***(A)** Data for test crickets that received PS+2 AS with the latter spaced 1 s, 30 s or 60 s apart. **(B)** Data for test crickets that received either no prior stimulation (untreated), AS followed by PS 0.5 min later (AS+PS) or PS followed by AS 0.5, 1, 3, or 10 min later (PS+AS). The data are depicted in Figure 2*.

We next evaluated the temporal limits for the association between the PS and 2 AS (Figure 2B). Two AS had no effect on aggression when presented shortly before the PS (U-tests compared to no stimulus: level, *p* = 0.300, duration, *p* = 0.6475; Figure 2, Table 2), but effectively suppressed aggression when delivered 0.5 or 1 min after the PS (e.g., for 1min, U-tests compared to none: level, *p* < 0.0001; duration, *p* < 0.0001; Figure 2, Table 2). In comparison, a PS-AS latency of 3 min was far less effective (U-tests compared to none: level, *p* = 0.079, duration, *p* = 0.061) and a latency of 10 min no longer affected aggression.

**Figure 3:**
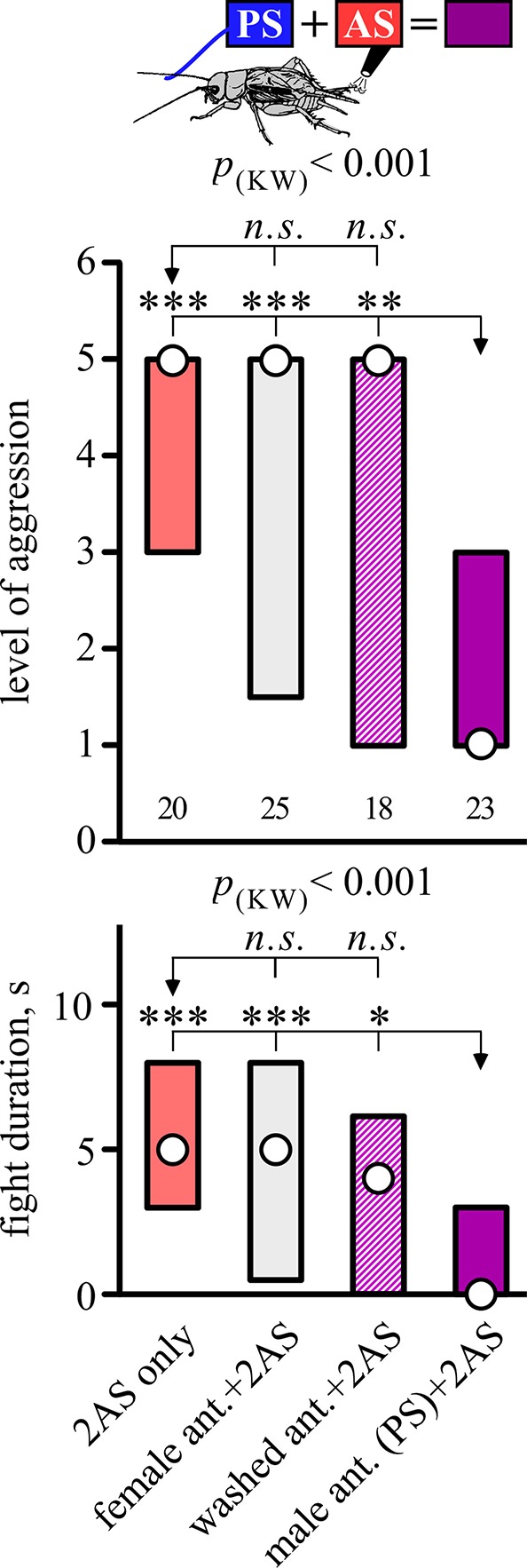
**Requirement for a male antenna as the PS**. Aggressiveness of crickets matched against a standard hyper-aggressive opponent 10 min after a stimulation (top, standard stimulus regime; middle, level of aggression; bottom, fight duration in s.). Circles: median, boxes: interquartile range, *n* is given below the bars. The animals received, from left to tight the AS only (red bar), or AS preceded by antennal stimulation with either a female antenna (gray bar), a washed antenna (hatched purple bar) or a male antenna (standard PS, purple bar). Significant differences for the data sets are given as *p*-value from Kruskal-Wallis tests (*p*_(kw)_), and differences between specific groups (arrows) by asterisks from Dunn's multiple comparisons test (^*^p < 0.05, ^**^p < 0.01, ^***^p < 0.001, *n.s*. not significant).

Interestingly, the suppressing effect of AS was only evident when preceded by PS with a fresh male antenna, but not if preceded by stimulation with a washed male antenna (Dunn's multiple comparisons test: level, *p* < 0.01, duration, *p* < 0.05; Figure 3, Table 3), or stimulation with a female antenna (Dunn's multiple comparisons test: level, *p* < 0.001, duration, *p* < 0.001), the courtship releasing stimulus (cf. Rillich and Stevenson, 2015). Supporting this, the level and duration of aggression after AS only, was not significantly different to that for animals that received AS preceded by stimulation with a washed or female antenna.

**Table 3:** **Table giving fight level and duration of aggression of aggression for fights of test crickets against standard hyper-aggressive opponents: median (50%), IQR (25, 75%), minimum (min.), maximum (max.), sample size (*n*) and statistics values from Kruskal-Wallis tests (*H, p*)**.

**Group**	**Fight level**						**Fight duration, s**					**Statistics**	
	**n**	**min**	**25%**	**50%**	**75%**	**max**	**min**	**25%**	**50%**	**75%**	**max**	***H***	***p***
male PS+2AS	23	1	1	1	3	5	0	0	0	3	6	Level:	
washed PS+2AS	18	1	1	5	5	6	0	0	4	6.25	16	21.76	0.001
female PS+2AS	25	1	1.5	5	5	6	0	0.5	5	8	44	Duration:	
2AS	20	1	3	5	5	6	0	3	5	8	27	23.76	0.001

*The test crickets received either the standard PS with a male antenna (male PS), PS with a washed-, or a female antenna followed by 2AS, or 2AS only. The data are depicted in Figure 3*.

### Effects of amine receptor blockers

**Figure 4:**
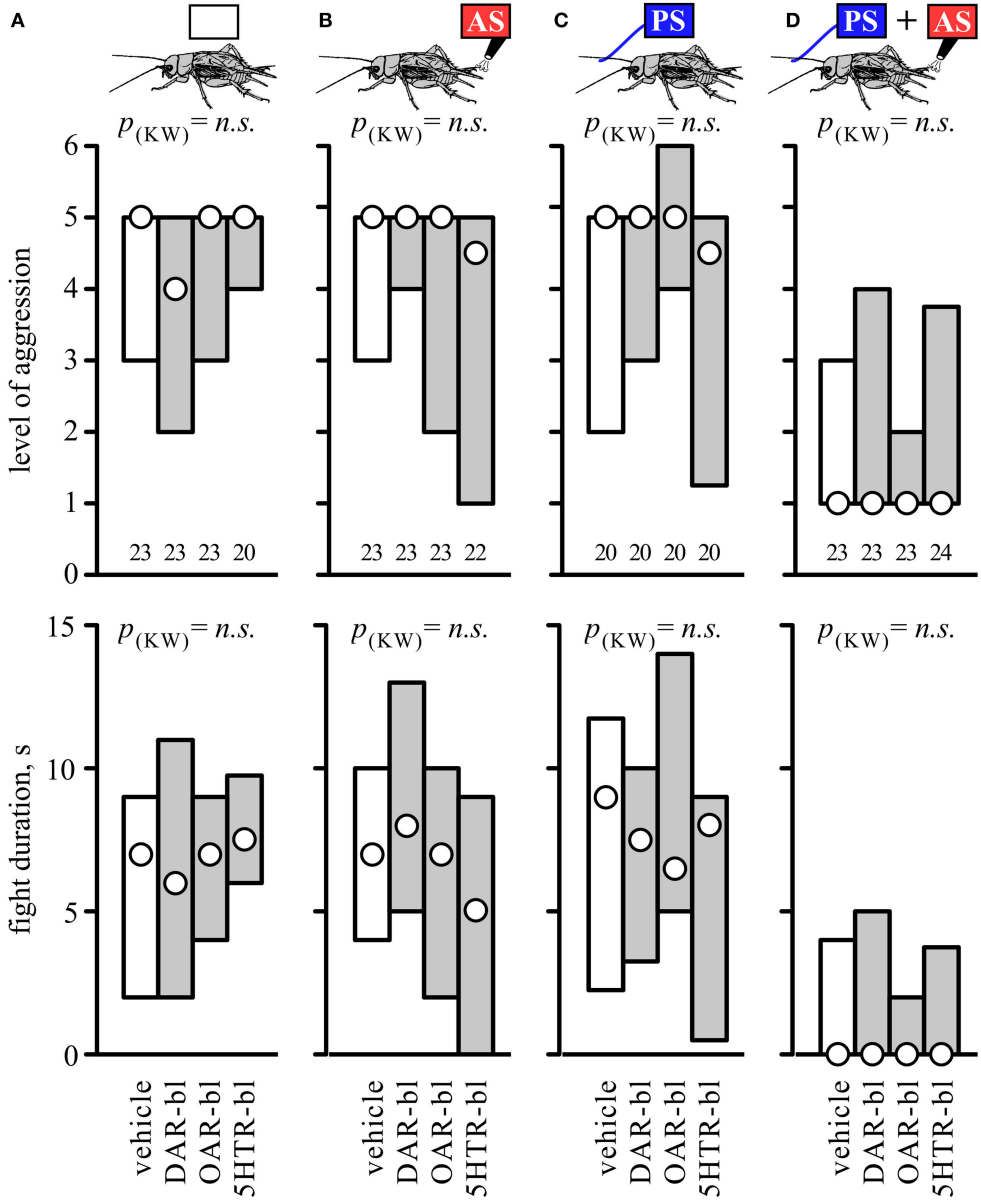
**Influence of amine receptor blockers**. Bar charts giving the aggressiveness of test crickets (top, stimulus regime; middle, level of aggression; bottom, fight duration, s) matched against standard hyper-aggressive opponents 10 min after different stimuli and drug treatments: **(A)** No prior sensory stimulation, **(B)** 2 AS alone, **(C)** PS alone, **(D)** 2 AS preceded by PS (circles: median, boxes: interquartile range, *n* is given below the bars). In each case the crickets were pretreated with either vehicle (1% DMSO in saline, white bars) or blockers selective for dopamine, octopamine or serotonin receptors (gray bars: DAR-bl, OAR-bl, 5HT-bl). Kruskal-Wallis tests gave no significant differences for the vehicle and drug treatments (*p*_(kw)_ = *n.s*.).

To investigate whether biogenic amines influence the responses to AS, PS or a combination of both, separate groups of socially naive crickets were first treated with antagonists for different amine receptors. Their aggressive performances against standard hyper-aggressive opponents were then evaluated after either no further treatment (Figure 4A), after 2 AS alone (Figure 4B), after PS alone (Figure 4C), or after PS directly followed by 2 AS (Figure 4D). These experiments showed that the level of aggression and fight duration were not significantly affected in crickets treated with vehicle (1% DMSO in saline, Figure 4, Table 4), the dopamine receptor blocker fluphenazine (DAR-bl), the octopamine receptor blocker epinastine (OAR-bl) or a cocktail of the serotonin receptor blockers methiothepin and ketanserin (5HTR-bl), irrespective of whether the animals received no sensory stimulation (Kruskal-Wallace-test: level, *p* = 0.6377, duration, *p* = 0.792), prior 2 AS (Kruskal-Wallace-test: level, *p* = 0.4149, duration, *p* = 0.2505), prior PS (Kruskal-Wallace-test: level, *p* = 0.2936, duration, *p* = 0.508), or PS followed by 2 AS (Kruskal-Wallace-test: level, *p* = 0.9089, duration, *p* = 0.8771).

**Table 4:** **Table giving fight level and duration of aggression of aggression for fights of test crickets against standard hyper-aggressive opponents: median (50%), IQR (25, 75%), minimum (min.), maximum (max.), sample size (*n*) and statistics values from Kruskal-Wallis tests (*H, p*)**.

**Group**	**Fight level**						**Fight duration, s**					**Statistics**	
	**n**	**min**	**25%**	**50%**	**75%**	**max**	**min**	**25%**	**50%**	**75%**	**max**	***H***	***P***
DMSO none	23	1	3	5	5	6	0	2	7	9	61	Level:	
DAR-bl none	23	1	2	4	5	6	0	2	6	11	16	1.697	0.638
OAR-bl none	23	1	3	5	5	6	0	4	7	9	18	Duration:	
5HTR-bl none	20	1	4	5	5	6	0	6	7.5	9.75	25	1.038	0.792
DMSO 2AS	23	1	3	5	5	6	0	4	7	10	17	Level:	
DAR-bl 2AS	23	1	4	5	5	6	0	5	8	13	29	2.583	0.415
OAR-bl 2AS	23	1	2	5	5	6	0	2	7	10	29	Duration:	
5HTR-bl 2AS	22	1	1	4.5	5	6	0	0	5	9	14	4.103	0.251
DMSO PS	20	1	2	5	5	6	0	2.25	9	11.75	21	Level:	
DAR-bl PS	20	1	3	5	5	6	0	3.25	7.5	10	26	3.718	0.294
OAR-bl PS	20	1	4	5	6	6	0	5	6.5	14	20	Duration:	
5HTR-bl PS	20	1	1.25	4.5	5	6	0	0.5	8	9	21	2.323	0.508
DMSO PS+A2S	23	1	1	1	3	5	0	0	0	4	7	Level:	
DAR-bl PS+2AS	23	1	1	1	4	5	0	0	0	5	7	0.545	0.909
OAR-bl PS+2AS	23	1	1	1	2	5	0	0	0	2	6	Duration:	
5HTR-bl PS+2AS	24	1	1	1	3.75	6	0	0	0	3.75	12	0.683	0.877

*Test crickets were pre-treated with either vehicle (1% DMSO in saline), or selective blockers for dopamine, octopamine or serotonin receptors (DAR-, OAR-, 5HTR-blockers respectively and received in addition either no prior stimulation (none), 2 AS, the PS or PS+2AS. The data are depicted in Figure 4*.

### Effect of blocking nitric oxide (NO) production

**Figure 5:**
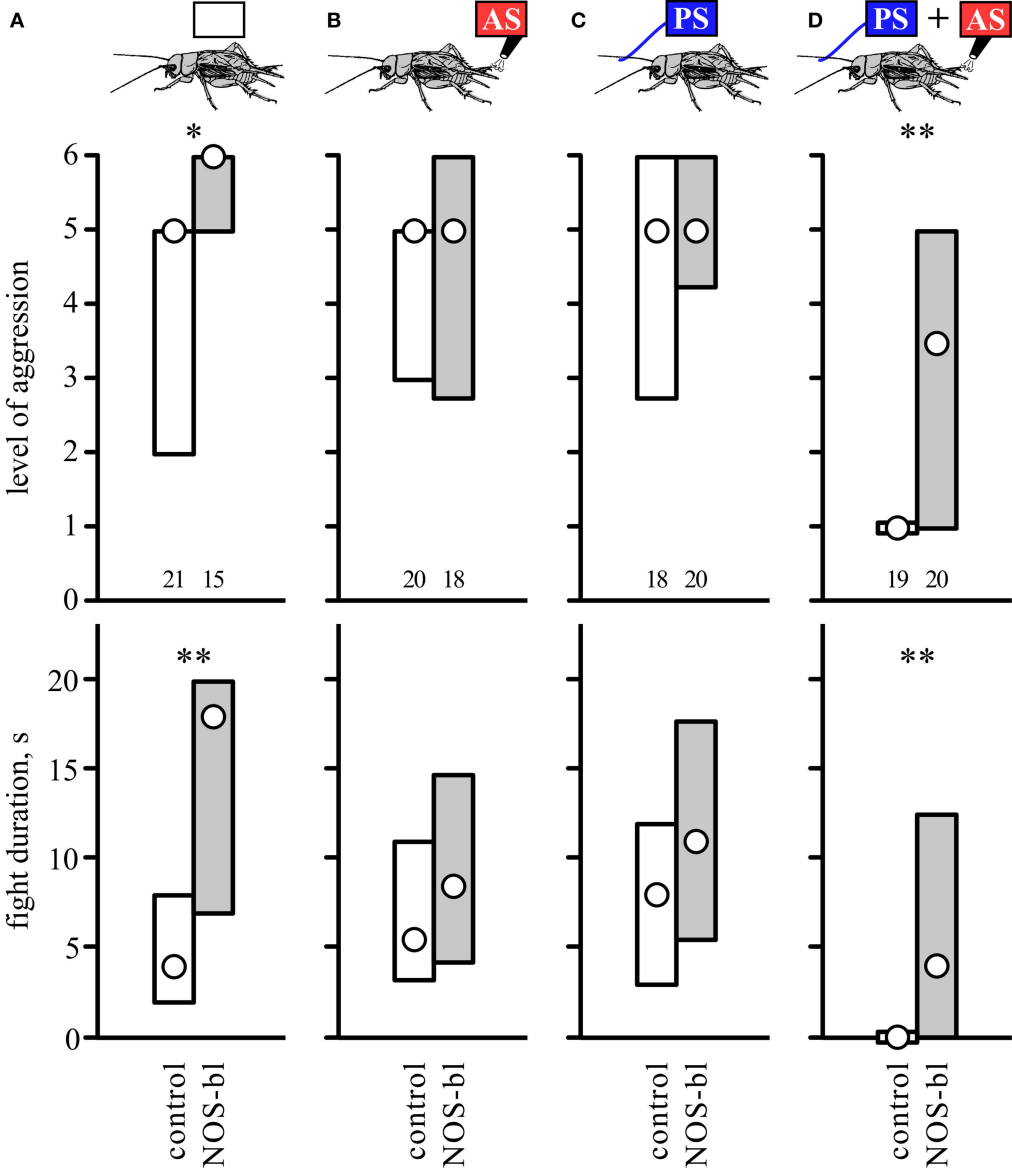
**Influence of nitric oxide synthesis blocker**. Bar charts giving the aggressiveness of test crickets (top, stimulus regime; middle, level of aggression; bottom, fight duration, s) matched against standard hyper-aggressive opponents 10 min after different stimuli and drug treatments: **(A)** No prior sensory stimulation, **(B)** 2 AS alone, **(C)** PS alone, **(D)** 2 AS preceded by PS (circles: median, boxes: interquartile range, *n* is given below the bars). In each case the crickets were pretreated with either the competitive nitric oxide synthase inhibitor LNAME (NOS-bl, gray bars) or its non-effective enantiomer (DNAME, control, white bars). Significant differences between LNAME treated and corresponding DNAME groups are indicated (U-test: ^*^p < 0.05, ^**^p < 0.01).

Pre-treatment with the competitive nitric oxide synthase (NOS) inhibitor N-nitro-L-arginine (LNAME) had profound effects on aggression (Figure 5, Table 5; test regime as in Figure 4). Firstly, and confirming our earlier findings (Stevenson and Rillich, 2015), socially naive crickets with blocked NO-production (LNAME) that received no sensory stimulation, escalated to higher levels and fought longer than control crickets that received the non-effective enantiomer DNAME (U tests: level, *p* = 0.0093, duration, *p* = 0.0067, Figure 5A). This difference between DNAME and LNAME treated crickets was, however, no longer evident in groups that received either AS or PS only (Figures 5B,C, statistics in Table 5). This is unlikely to be an effect of the AS or PS, since the level and duration of aggression in these groups is not significantly difference to the unstimulated group, regardless of whether treated with LNAME or DNAME (Kruskal-Wallace-test, LNAME: level, *p* = 0.20, duration, *p* = 0.25, DNAME: level, *p* = 0.14, duration, *p* = 0.37). More pertinent to the current study, while 2 AS preceded by the PS again essentially abolished the expression of aggression in control crickets (DNAME, median level 1, IQR 1-1, *n* = 19), the effect of paired PS-AS was considerably diminished in crickets with blocked NOS (LNAME: median level 3.5, IQR 1–5, *n* = 20), so that they were considerably more aggressive than DNAME treated control crickets (U tests: level, *p* = 0.0079; duration, *p* = 0.0061). In fact, while none of the DNAME treated crickets exhibited aggressive behavior after experiencing 2 AS preceded by the PS, half of the LNAME-treated crickets with the same experiences exhibited aggressive behavior (50% threat display, 35% physical fights, *n* = 20) toward standard hyper-aggressive opponents. We conclude that the gaseous modulator NO is necessary for the aggression suppressing effect of aversive stimulation when experienced in an aggressive behavioral context.

**Table 5:** **Table giving fight level and duration of aggression of aggression for fights of test crickets against standard hyper-aggressive opponents: median (50%), IQR (25, 75%), minimum (min.), maximum (max.), sample size (*n*) and statistics values from Mann-Whitney U-tests (*U, p*)**.

**Group**	**Fight level**						**Fight duration, s**					**Statistics**		
	**n**	**min**	**25%**	**50%**	**75%**	**max**	**min**	**25%**	**50%**	**75%**	**max**		***U***	***P***
DNAME none	21	1	2	5	5	6	0	2	4	8	21	Level:	81	0.009
LNAME none	15	1	5	6	6	6	0	7	18	20	26	Duration:	67	0.008
DNAME 2AS	20	1	3	5	5	6	0	3.25	5.5	11	24	Level:	176	0.94
LNAME 2AS	18	1	2.75	5	6	6	0	4.25	8.5	14.75	24	Duration:	148	0.355
DNAME PS	18	1	2.75	5	6	6	0	3	8	12	21	Level:	165	0.651
LNAME PS	20	1	4.25	5	6	6	0	5.5	11	17.75	35	Duration:	147	0.231
DNAME PS+2AS	19	1	1	1	1	5	0	0	0	1	10	Level:	105	0.008
LNAME PS+2AS	20	1	1	3.5	5	6	0	0	4	12.5	71	Duration:	101	0.006

*Test crickets were pre-treated with the competitive nitric oxide synthase inhibitor (LNAME) or its non-effective enantiomer DNAME as control and received in addition either no prior stimulation (none), 2 AS, the PS or PS+2AS. The data are depicted in Figure 5*.

### Effect of the nitric oxide donor SNAP

**Figure 6:**
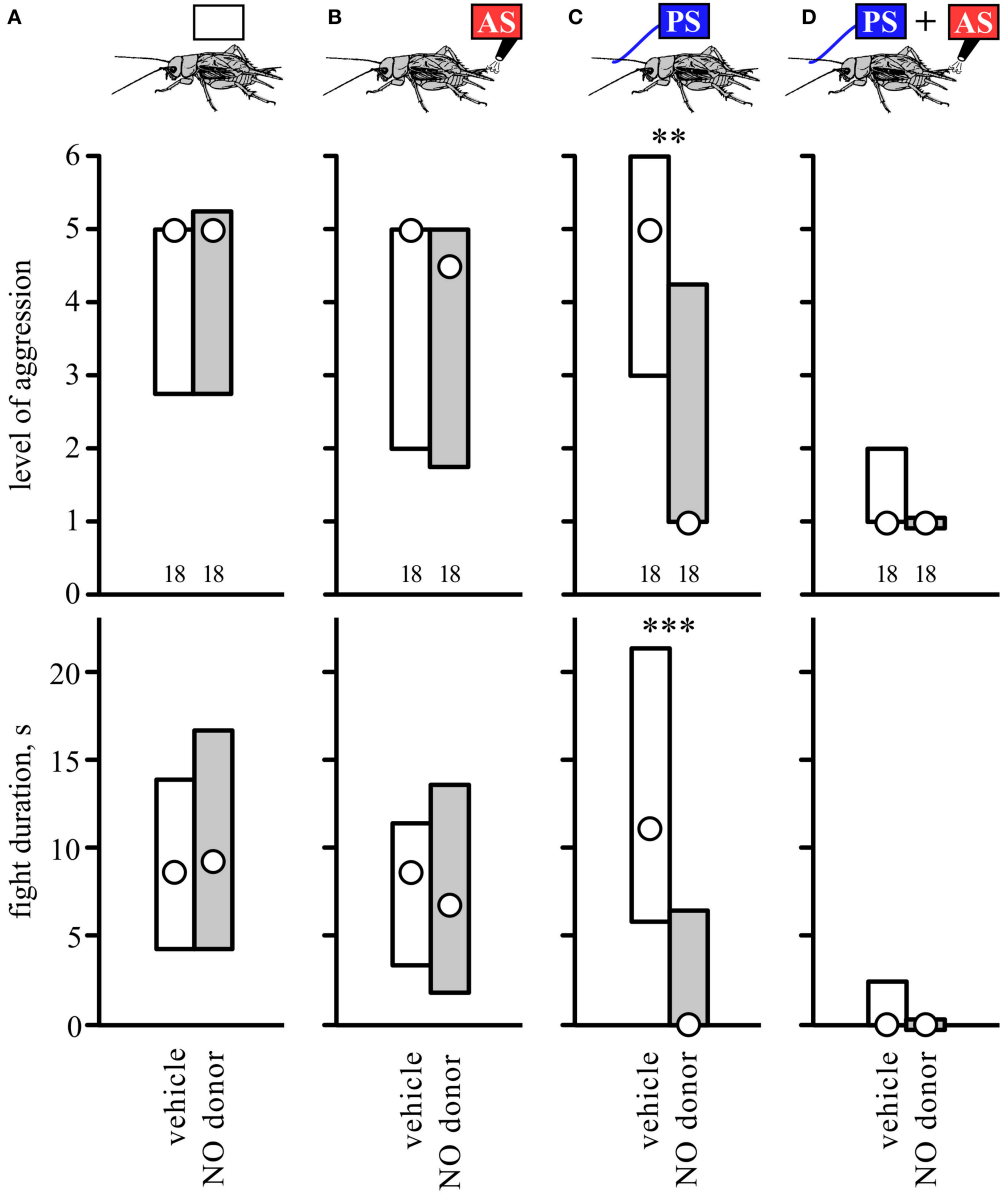
**Influence of nitric oxide donor SNAP**. Bar charts giving the aggressiveness of test crickets (top, stimulus regime; middle, level of aggression; bottom, fight duration, s) matched against standard hyper-aggressive opponents 10 min after different stimuli and drug treatments: **(A)** No prior sensory stimulation, **(B)** 2 AS alone, **(C)** PS alone, **(D)** 2 AS preceded by PS (circles: median, boxes: interquartile range, *n* is given below the bars). In each case the crickets were pretreated with either the NO donor SNAP (gray bars) or vehicle (insect saline, white bars). Significant differences between SNAP treated and corresponding vehicle groups are indicated (U-test: ^**^p < 0.01, ^***^p < 0.001).

We next tested whether the NO-donor SNAP could substitute for the AS. Since SNAP leads to a reduction in aggression on its own at a dosage of 5.0 mM (Stevenson and Rillich, 2015 and Figure S2), we selected to use the lower concentration of 1.0 mM where this effect is not evident (Stevenson and Rillich, 2015 and Figure S2). Confirming this, SNAP had no clear effect on the aggressiveness of untreated crickets compared to controls that received vehicle only when matched against standard hyper-aggressive opponents (Figure 6A, Table 6). Similarly, SNAP had no influence on the aggressiveness of crickets that received prior AS alone (Figure 6B, compare with Figure 1A). However, SNAP had clear effects on how the animals fought after receiving the PS alone. Whereas untreated- and vehicle-treated crickets tended to be more aggressive after receiving the PS only (Figures 1B, 6C), those that received SNAP were significantly less aggressive after experiencing the PS both in terms of the escalation level and duration of the fights (U tests: level, *p* = 0.0014; duration, *p* = 0.0004, Table 6). In fact, 11 out of 18 of these SNAP-treated crickets (61%) behaved exactly as losers in that they retreated immediately on confronting an opponent (level 1), compared to 11% immediate retreats for vehicle-treated control animals after receiving the PS (*n* = 18, Chi-square test: Chi-value = 9.76, *p* = 0.0017). Finally, crickets that received PS followed by the AS were essentially non-aggressive, irrespective of whether they received no-drug, vehicle, or SNAP (Figure 6D, compare with Figure 1B). Taken together, the results suggest that the NO-donor SNAP can substitute for the AS and suppress aggression, but only when the animal is primed for aggression.

**Table 6:** **Table giving fight level and duration of aggression of aggression for fights of test crickets against standard hyper-aggressive opponents: median (50%), IQR (25, 75%), minimum (min.), maximum (max.), sample size (*n*) and statistics values from Mann-Whitney U-tests (*U, p*)**.

**Group**	**Fight level**						**Fight duration, s**					**Statistics**		
	**n**	**min**	**25%**	**50%**	**75%**	**max**	**min**	**25%**	**50%**	**75%**	**max**		***U***	***p***
Saline none	18	1	2.75	5	5	6	0	3.5	7	11.25	15	Level:	161	0.988
SNAP none	18	1	2.75	5	5.25	6	0	3.5	7.5	13.5	38	Duration:	147	0.643
saline 2AS	18	1	2	5	5	6	0	2.75	7	9.25	21	Level:	150	0.725
SNAP 2AS	18	1	1.75	4.5	5	6	0	1.5	5.5	11	17	Duration:	149	0.688
saline PS	18	1	3	5	6	6	0	4.75	9	17.25	61	Level:	66	0.0014
SNAP PS	18	1	1	1	4.25	6	0	0	0	5.25	17	Duration:	57	0.001
saline PS+2AS	18	1	1	1	2	5	0	0	0	2	6	Level:	136	0.327
SNAP PS+2AS	18	1	1	1	1	4	0	0	0	0	5	Duration:	134	0.267

*Test crickets were pre-treated with either vehicle (saline) or the NO donor SNAP and received in addition either no stimulation (none), 2 AS, the PS or PS+2AS. The data are depicted in Figure 6*.

## Discussion

In this paper, we investigated whether experiencing repeated potentially aversive tactile stimuli in crickets can induce the social defeat syndrome, typified by a state of prolonged submissiveness that normally only occurs after losing a fight with a conspecific male (Hsu et al., 2006). Although we speculated that aversive stimulation alone might lead to reduced aggressiveness (Stevenson and Rillich, 2015), repeated wind stimulation of the abdominal cercal organs had no influence on a cricket's propensity to fight (Figure 1A). By itself, therefore, potentially aversive stimuli (AS), or simple handling as claimed in *Drosophila* (Trannoy and Kravitz, 2015), do not appear to reduce aggression in crickets. However, when paired with an aggression-priming stimulus (PS: antennal stimulation with another male's antennae), AS led to the establishment of the submissive behavioral state typical for social defeat (Figure 1B). We would like to stress that this effect is not dependent on either of these stimuli eliciting a motor response. While the PS often elicits threat display (47%), it was equally effective at suppressing aggression when paired with the AS in animals that did not show the threat display. Furthermore, the intensity of the AS was set to below that required to elicit escape walking jumps or runs (cf. Gras and Hörner, 1992; Oe and Ogawa, 2013; Fukutomi et al., 2015), and aggression was always evaluated 10 min after experiencing these stimuli. Notably, prior stimulation with a washed male, or female antennae was not effective (Figure 3), indicating that male cuticular pheromones (cf. Iwasaki and Katagiri, 2008) are a necessary component of the aggression-priming stimulus (see also Sakura and Aonuma, 2013; Andrews et al., 2014; Rillich and Stevenson, 2015). Furthermore, the PS was only effective when it preceded the AS, and the latter was only effective when applied within 1 min after the PS (Figure 2B). Thus, potentially detrimental sensory stimuli can only acquire the attribute of being an aversive agonistic signal, which can effectively induce submissiveness, when the animal is primed to exhibit aggressive behavior by antennal contact with another male. That this can be implemented by wind stimulation of the cerci shows that the assessment of opponent actions for the decision to flee need not be based on classical agonistic signals, such as threats and bites, normally associated with aggression. The cercal mechanosensory system transduces low frequency air movements near the animal's body and is currently thought to function as a low-frequency, near-field extension of the animal's auditory system that can provide the animal with relevant information about its environment, and affect many behaviors in addition to escape (Jacobs et al., 2008). We propose that any potentially detrimental stimulus can acquire the attribute of an aversive agonistic signal when experienced in an aggressive context. This fits with the idea that insects can use “short-cuts” (Wehner, 1981), i.e., readily identifiable, characterizing features to recognize mates, prey, or predators. For example, rather than evaluating the qualities of a variety of complex agonistic signals with different modalities, crickets appear to assess any signal as aversive when perceived in an aggressive context, and need only to equate its net sensory impact.

The fact that a single AS paired with the PS is far less effective at inducing a subordinate status than two or more AS (Figure 1B), conforms to the hypothesis that crickets add up their opponent's aversive actions during fighting and flee when the sum accrued exceeds some critical level (Rillich et al., 2007; Stevenson and Rillich, 2015), as originally envisaged by the Cumulative Assessment Model of Payne (1998). Furthermore, the observation that the effectiveness of two successive AS waned with increasing inter-stimulus interval, and became ineffective when spaced by more than 1 min (Figure 2B), suggests the presence of a sensory memory (cf. Menzel, 2001) for each AS which fades after 60 s. Thus, as an extension to Payne's (1998) Cumulative Assessment Model concept, the subordinate status in crickets is established by the frequency of opponent's aversive actions experienced during a limited time period, rather than the simple sum.

The depression of aggression in subordinates after losing is suggested to reflect a memory of past fighting experience (Yurkovic et al., 2006; Trannoy and Kravitz, 2015). Our finding that AS must be paired with prior PS to induce submissiveness should not, however, be confused with forward aversive conditioning paradigms for establishing negative memories (review: Tedjakumala and Giurfa, 2013). In classical associative aversive learning, for example in honey bees, forward-pairing of an odor (the conditioned stimulus, CS) with an electric shock (the unconditioned stimulus, US) results in bees learning this contingency and thereafter extend their sting in response to the previously punished odor (Vergoz et al., 2007). By analogy, the AS would correspond to the US, but since it fails to induce submissiveness on its own, it is not acting like a US. Similarly, since the PS alone also fails to depress aggression, the situation is not comparable to backward conditioning. Hence, the temporal association formed between the PS and AS is not equivalent to classical aversive conditioning.

Considering the key roles of biogenic amines in modulating aggression (reviews: Nelson and Trainor, 2007; Kravitz and Fernandez, 2015; Stevenson and Rillich, 2016), sensory perception (review: Scheiner et al., 2006) and associative learning (review: Giurfa, 2006) we were surprised to find that a wide range of amine-receptor antagonists failed to abolish the aggression suppressing effects of AS paired with the PS (Figure 4). That the blockers had no clear effect on fights of otherwise non-stimulated crickets (Figure 4A) is not so surprising considering that amines are not essential for the initiation of aggression *per se* (Rillich and Stevenson, 2015). While the octopamine receptor blocker epinastine can lead to a just significant reduction of aggression in naïve crickets (*p* < 0.05, see Stevenson et al., 2005; Rillich et al., 2011), this is not always evident (e.g., Rillich and Stevenson, 2011). This reflects the role of octopamine as a neuromodulator that acts primarily to mediate the aggression promoting effect of diverse experiences including flying, winning, and resource possession, effects that are each specifically blocked by epinastine (Stevenson et al., 2005; Rillich and Stevenson, 2011, 2015; Rillich et al., 2011; Stevenson and Rillich, 2016). However, we did expect that epinastine would influence aggression at least in crickets that received prior PS plus AS. In submissive crickets at least, the PS has an aggression enhancing effect, that depends on octopamine and is blocked selectively by epinastine (Rillich and Stevenson, 2015). Similarly, octopamine is necessary for *Drosophila* males to coordinate sensory cue information presented by a second male, allowing them to respond with aggressive behavior (Certel et al., 2007). Furthermore, to be effective, an unwashed male antenna is required as the PS (Figure 3). This implicates male contact pheromone as a necessary component of the PS (see also Rillich and Stevenson, 2015). In *Drosophila* pheromonal signals detected by chemosensory neurons enhance male aggression *via* activation of specific octopaminergic neurons (Certel et al., 2007; Andrews et al., 2014). If this holds for crickets, then the PS should no longer be effective in epinastine treated crickets, and accordingly crickets that received the PS+AS would not become submissive. As it stands, however, our current data indicate that amines play no major role in forming the association between the PS and AS, which stands in marked contrast to associative learning, where they have manifold influences (Giurfa, 2006).

Contrary to aminergic receptor blockers, the NO synthesis inhibitor LNAME, but not its biologically inactive enantiomer DNAME, completely abolished the establishment of submissiveness by paired PS-AS stimulation (Figure 5). Since the NOS inhibitor LNAME can alone lead to an increase in aggression (Figure 5), one might argue that NO acts primarily to decrease the propensity to fight. While this cannot be entirely refuted, it seems to be relatively unlikely in view of the effect of the NO donor SNAP. Although SNAP at high concentrations (5 mM) can depress aggression on its own (Figure S2; see also Stevenson and Rillich, 2015) this does not occur at the lower concentration (1 mM) used in this study. At this lower concentration, the NO donor SNAP induced submissive behavior typical for the social defeat syndrome, only after the animals received the PS, and not in those that were unstimulated or received the AS alone. This suggests that the gaseous modulator NO acts selectively by substituting for AS in our paradigm. Hence, we suggest that NO mediates the effects of the AS. Supporting this idea, PS + AS no longer induces the social defeat syndrome in LNAME treated crickets (Figure 5D) since the effect of the AS is blocked. This interpretation conforms with experiments indicating that potentially aversive agonistic signals of an opponent experienced during fighting promote the decision to flee and establishes the social defeat syndrome via the action of NO (Stevenson and Rillich, 2015). Future studies could test whether the timing of delivery of NO-donor relative to the PS follows the same temporal requirement for the AS and PS to induce the submissive behavioral status, but this would only be feasible with harnessed crickets.

At present, we can only speculate on the sequence of events leading to the induction of submissiveness via the action of NO. The most parsimonious explanation is that AS activates NOS only when experienced in an aggressive context. This is induced by antennal fencing in natural behavior, and experimentally by the PS. Thus, combined mechanical and male pheromonal stimulation appears necessary to gate activation of NOS by aversive afferent signals. Once released, NO could represent the aversive quality of the stimulus, and in sufficient quantities act to induce submissiveness. This fits with our hypothesis that crickets make the decisions to flee when the sum of an opponent's aversive actions surpass some threshold which leads to activation of the NO/cGMP pathway (Stevenson and Rillich, 2015, 2016, 2017). What we first need to know, however, is whether NOS is activated with each discrete aversive event, or first after experiencing some critical net sum. The architecture and actions of the NO signaling system of insect brains is well adapted for this task. Fine arbors that can release NO as a volume signal occur in all major integration centers (Kurylas et al., 2005; Ott et al., 2007). Beyond the role of NO/cGMP as an essential pathway in long-term memory (Menzel and Muller, 1996), cGMP-dependent protein kinase is required for a seconds long working memory for goal-driven behavior in *Drosophila* (Kuntz et al., 2012), a phenomenon that could conceivably underlie a cricket's ability to “add up” an opponent's aversive actions for the decision to flee.

Taken together, our findings illustrate that the seemingly complex social decision to flee and the subsequent social defeat syndrome can be implemented by a comparatively simple mechanism. In crickets, the experience of antennal contact with a conspecific male (PS) establishes an aggressive behavioral context, during which subsequent sensory experiences acquire the quality of an aversive stimulus (AS), that can act to induce production of the neuromodulator NO. The fact that we can use simple stimuli as tools to set and swap a cricket's social status from submissive to aggressive (PS, Rillich and Stevenson, 2015) and back to submissive (PS+AS, this paper) opens new avenues for investigating the mechanisms underlying the decision to fight or flee in animal conflict. At present, we know of no other animal model system in which similar insights have been revealed. Despite many models for decision-making behavior, recent experimental findings casts doubt on the idea of evidence accumulation as a general decision-making mechanism even in humans (van Vugt et al., 2016). Even so, NO has numerous roles in synaptic integration and motor behavior of vertebrates (review: Del Bel et al., 2005), where for example inhibition of cGMP dependent protein kinase is required for retention of passive avoidance tasks in chicks (Edwards et al., 2002). NOS knockout has also revealed that NO acts to reduce aggression in mice, largely *via* interactions with serotonin (Nelson and Trainor, 2007). The exact behavioral role of NO in mammalian aggression remains, however, unclear. Given the relevance of social defeat for understanding depression and other psychiatric disorders in humans (Huhman, 2006; Hollis and Kabbaj, 2014), and the still limited knowledge of proximate causes, our findings encourage deeper investigations of the behavioral roles of NO in mammalian decision making and depression-like behavioral syndromes.

## Author contributions

Conceived and designed the experiments: JR and PS. Performed the experiments: JR. Analyzed the data: JR and PS. Contributed reagents/materials/analysis tools: PS and JR. Wrote the paper: PS and JR.

## Funding

Support by the German Research Council (DFG) is greatly appreciated (Grants STE 714/4-1 and RI 2728/2-1).

### Conflict of interest statement

The authors declare that the research was conducted in the absence of any commercial or financial relationships that could be construed as a potential conflict of interest.

## Supplementary material

Figure S1 **Dosage dependent effect of the NOS blocker LNAME**. Bar charts giving the aggressiveness of test crickets (top, stimulus regime; middle, level of aggression; bottom, fight duration, s) matched against standard hyper-aggressive opponents 10 min after different treatments: **(A)** No prior sensory stimulation, **(B)** 2 AS preceded by PS (circles: median, boxes: interquartile range, *n* is given below the bars). The crickets received either vehicle (20 μl insect saline, white bar), or LNAME (20 μl of 1, 5 or 10 mM, gray bars). Significant differences for the data sets are given as *p*-value from Kruskal-Wallis tests (*p*_(kw)_), asterisks indicate significant differences to saline treatment (Dunn's multiple comparisons test: ^*^*p* < 0.05).

Figure S2 **Dosage dependent effect of the NO donor SNAP**. Bar charts giving the aggressiveness of test crickets (top, stimulus regime; middle, level of aggression; bottom, fight duration, s) matched against standard hyper-aggressive opponents 10 min after different treatments: **(A)** No prior sensory stimulation, **(B)** 2 PS (circles: median, boxes: interquartile range, *n* is given below the bars). The crickets received either vehicle (20 μl insect saline, white bar), or SNAP (20 μl of 0.1, 1.0, or 5.0 mM gray bars). Significant differences for the data sets are given as *p*-value from Kruskal-Wallis tests (*p*_(kw)_), asterisks indicate significant differences to saline treatment (Dunn's multiple comparisons test: ^*^*p* < 0.05, ^**^*p* < 0.01, ^***^*p* < 0.001).

Table S1 **Table giving drug names, their abbreviation (Abbr.) and dosages as in text (mM/μl) and as weight (μg) and weight/animal body weight (μg/g wt.), using mean body weight as a reference (1.35 g, standard deviation 0.23)**. The administered dosage gives only a limited indication of the concentration effective in the nervous system due to dilution in tissues and the permeability barrier presented by the ganglion sheath (cf. Schofield et al., 1984; Stevenson et al., 2005).
