# Supplementary material for: Losing without Fighting - Simple Aversive Stimulation Induces Submissiveness Typical for Social Defeat via the Action of Nitric Oxide, but Only When Preceded by an Aggression Priming Stimulus
Source: Front Behav Neurosci. 2017 Mar 22;11:50. doi: 10.3389/fnbeh.2017.00050 (PMC5360729; doi:10.3389/fnbeh.2017.00050)

**Supplementary Table 1.** Table giving drug names, their abbreviation (Abbr.) and dosages as in text (mM/µl) and as weight (µg) and weight/animal body weight (µg/g wt.), using mean body weight as a reference (1.35 g, standard deviation 0.23). Note that the administered dosage gives only a limited indication of the concentration effective in the nervous system due to dilution in tissues and the permeability barrier presented by the ganglion sheath (cf. Schofield, 1984, Stevenson et al., 2005).

| **applied drug** | **Abbr.** | **dosage**  **mM/µl** | **dosage**  **µg** | **rel. dosage**  **µg/g wt.** |
| --- | --- | --- | --- | --- |
| epinastine hydrochloride | OAR-bl | 10/20 | 57 | 42,2 |
| fluphenazine dihydrochloride | DAR-bl | 10/20 | 102 | 75,6 |
| ketanserin (+)-tartrate salt | 5HT-bl | 10/20 | 109 | 80,7 |
| methiothepin mesylate salt | 5HT-bl | 10/20 | 90,4 | 67,0 |
| N_ω_-Nitro-L-arginine methyl ester hydrochloride (LNAME) | NOS-bl | 10/20 | 53,8 | 39,9 |
| N_ω_-Nitro-D-arginine methyl ester hydrochloride (DNAME) | control | 10/20 | 53,8 | 39,9 |
| S-Nitroso-N-acetyl-DL-penicillamine (SNAP) | NO-donor | 1/20 | 4,4 | 3,3 |

Figure S1


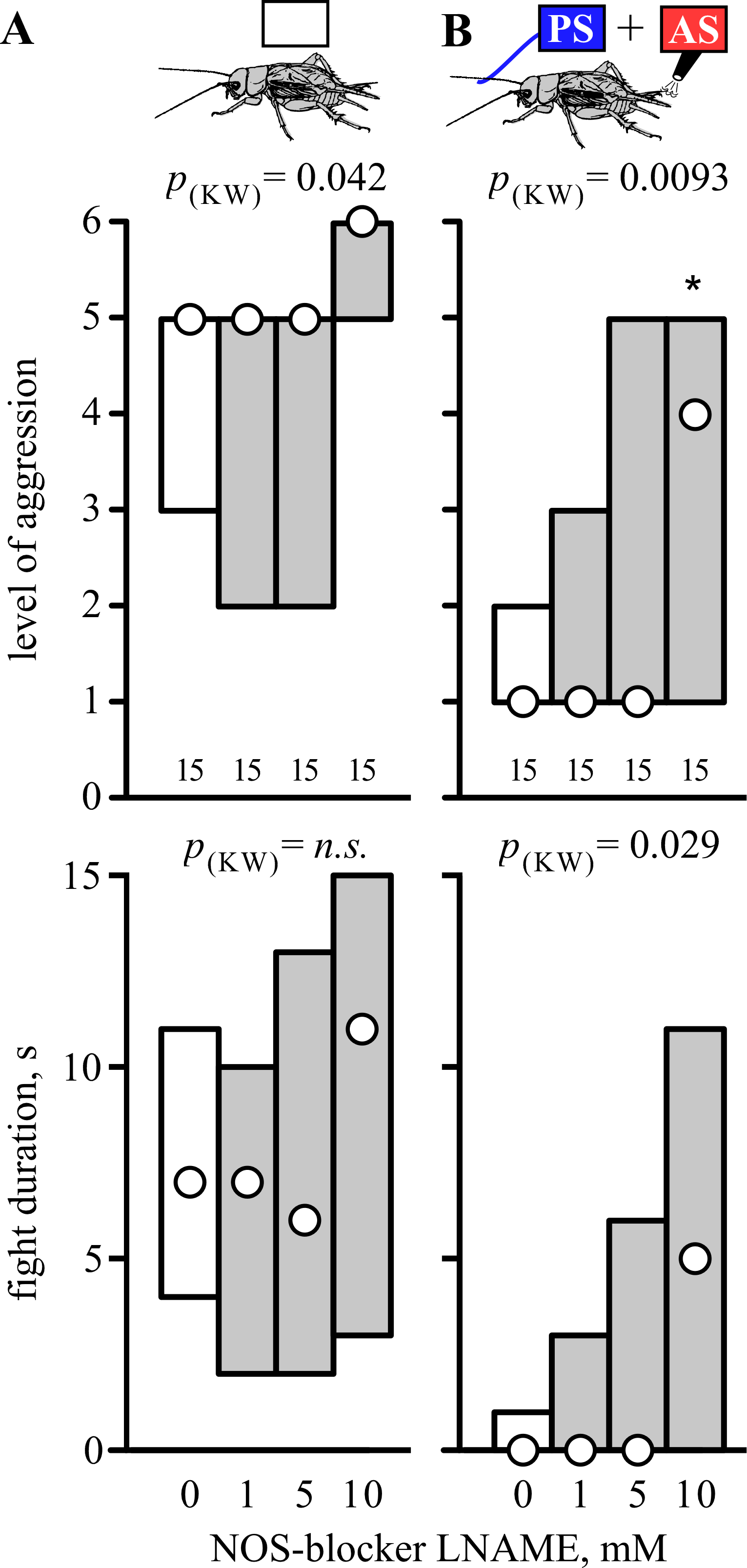


Figure S2


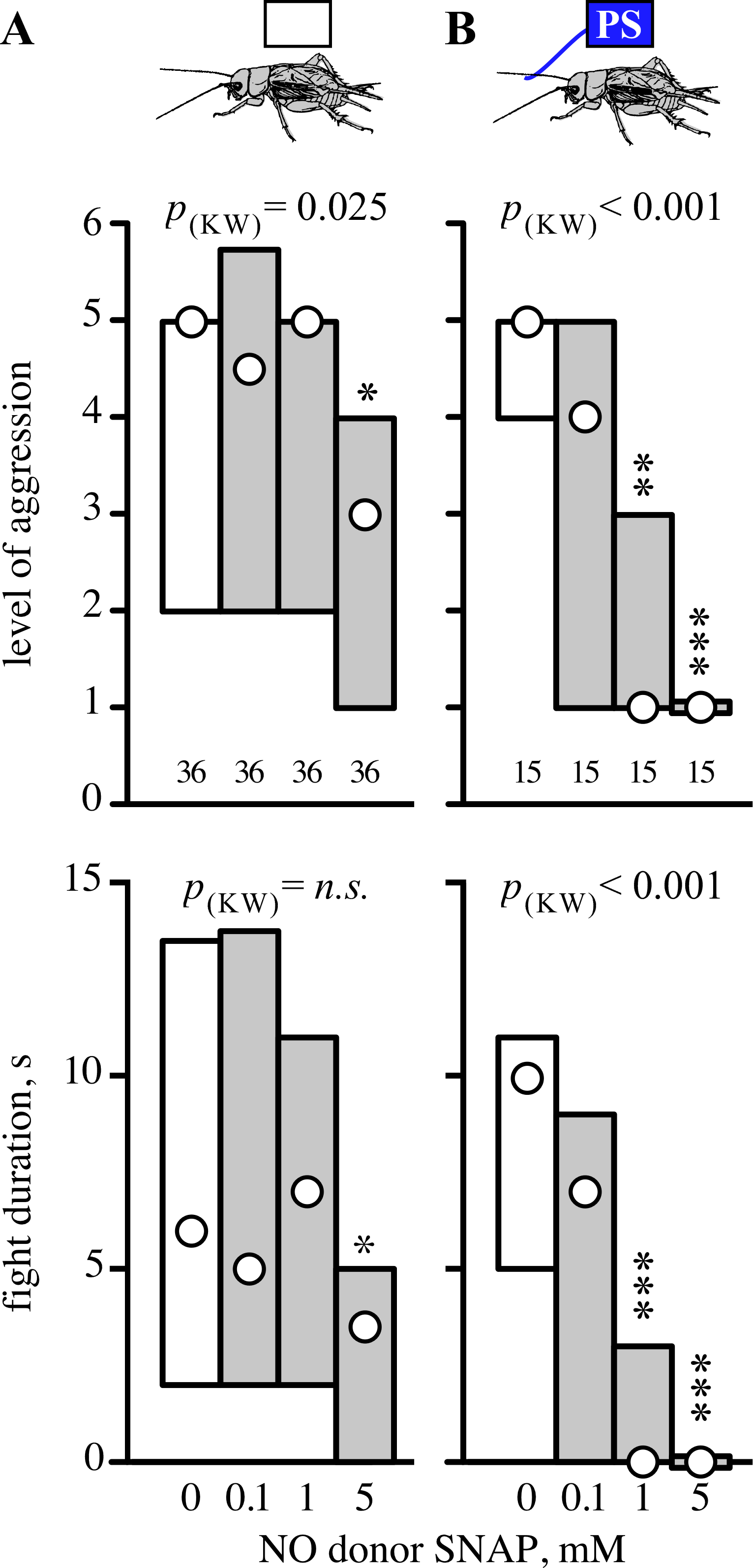

Supplement: Figure S1 — Dosage dependent effect of the NOS blocker LNAME. Bar charts giving the aggressiveness of test crickets (top, stimulus regime; middle, level of aggression; bottom, fight duration, s) matched against standard hyper-aggressive opponents 10 min after different treatments: (A) No prior sensory stimulation, (B) 2 AS preceded by PS (circles: median, boxes: interquartile range, n is given below the bars). The crickets received either vehicle (20 μl insect saline, white bar), or LNAME (20 μl of 1, 5 or 10 mM, gray bars). Significant differences for the data sets are given as p-value from Kruskal-Wallis tests (p(kw)), asterisks indicate significant differences to saline treatment (Dunn's multiple comparisons test: *p < 0.05). [file DataSheet1.docx]
